# Supplementary material for: Insect Body Defence Reactions against Bee Venom: Do Adipokinetic Hormones Play a Role?
Source: Toxins (Basel). 2021 Dec 23;14(1):11. doi: 10.3390/toxins14010011 (PMC8780464; doi:10.3390/toxins14010011)
Supplement: Supplementary file 1 [file toxins-14-00011-s001.zip › toxins-1499914-supplementary.pdf]

Table S1: List of plants blooming in the vicinity of the apiary in České Budějovice (CZ; 48° 58′ 31.924″ N, 14° 26′ 44.671 ″E; altitude 390 m) in June 2020.

**June 2020**	*Robinia pseudoacacia, Trifolium pratense, Crataegus, Sinapis alba, Phacelia tanacetifolia, Fagopyrum esculentum, Linum usitatissimum, Papaver rhoeas, Matricaria chamomilla, Paeonia sp., Jasminum, Viburnum sp., Iris sp., Centaurea sp., Tilia sp., Leucanthemum sp.*
